# A simple mortality prediction model for sepsis patients in intensive care

**DOI:** 10.1177/17511437221149572

**Published:** 2023-02-01

**Authors:** Hazem Koozi, Adina Lidestam, Maria Lengquist, Patrik Johnsson, Attila Frigyesi

**Affiliations:** 1 Department of Clinical Medicine, Anaesthesiology and Intensive Care, Lund University, Lund, Sweden; 2 Kristianstad Central Hospital, Anaesthesia and Intensive Care, Kristianstad, Sweden; 3 Skåne University Hospital, Intensive and Perioperative Care, Lund, Sweden; 4 Skåne University Hospital, Intensive and Perioperative Care, Malmö, Sweden

**Keywords:** Critical care, intensive care units, mortality, prognosis, risk adjustment, sepsis

## Abstract

**Background:**

Sepsis is common in the intensive care unit (ICU). Two of the ICU’s most widely used mortality prediction models are the Simplified Acute Physiology Score 3 (SAPS-3) and the Sequential Organ Failure Assessment (SOFA) score. We aimed to assess the mortality prediction performance of SAPS-3 and SOFA upon ICU admission for sepsis and find a simpler mortality prediction model for these patients to be used in clinical practice and when conducting studies.

**Methods:**

A retrospective study of adult patients fulfilling the Sepsis-3 criteria admitted to four general ICUs was performed. A simple prognostic model was created using backward stepwise multivariate logistic regression. The area under the curve (AUC) of SAPS-3, SOFA and the simple model was assessed.

**Results:**

One thousand nine hundred eighty four admissions were included. A simple six-parameter model consisting of age, immunosuppression, Glasgow Coma Scale, body temperature, C-reactive protein and bilirubin had an AUC of 0.72 (95% confidence interval (CI) 0.69–0.75) for 30-day mortality, which was non-inferior to SAPS-3 (AUC 0.75, 95% CI 0.72–0.77) (*p* = 0.071). SOFA had an AUC of 0.67 (95% CI 0.64–0.70) and was inferior to SAPS-3 (*p* < 0.001) and our simple model (*p* = 0.0019).

**Conclusion:**

SAPS-3 has a lower prognostic value in sepsis than in the general ICU population. SOFA performs less well than SAPS-3. Our simple six-parameter model predicts mortality just as well as SAPS-3 upon ICU admission for sepsis, allowing the design of simple studies and performance monitoring.

## Introduction

### Background

Sepsis is one of the most frequent diagnoses in intensive care units (ICUs), accounting for around 30% of all ICU admissions in Europe.^1,2^ It is also one of the leading causes of death in the world.^3^ The criteria for sepsis are suspected or documented infection and an acute increase of two Sequential Organ Failure Assessment (SOFA) points or more.^4^

The high incidence and mortality of sepsis make reliable and easy ICU prognostication of septic patients crucial. The Acute Physiologic and Chronic Health Evaluation II (APACHE II) and the Simplified Acute Physiology Score 3 (SAPS-3) are two of the most commonly used models for the assessment of illness severity and prediction of outcomes in the ICU.^5^ While SAPS-3 has been shown to have high sensitivity and specificity, it is developed for a mixed ICU cohort.^6^ However, the ICU population is heterogeneous, and neither SAPS-3 nor APACHE scores are designed to estimate mortality rates for any specific ICU subgroup. Both SAPS-3 and APACHE II have demonstrated good mortality prediction in postoperative surgical patients.^7^ Studies have also shown SAPS-3 to have good discriminatory power and calibration, although overestimating hospital mortality in a mixed ICU population.^8–12^ However, studies have indicated that SAPS-3 is challenging to use in clinical practice due to its many variables.^13^ The SOFA score has previously demonstrated good prognostic accuracy in predicting hospital mortality in patients with suspected infection admitted to the ICU. Still, it is mainly intended for measuring and monitoring organ failure.^14,15^

### Objectives

This study aimed to assess the prognostic performance regarding mortality of SAPS-3 and SOFA score for patients with sepsis (according to Sepsis-3) admitted to the ICU and to find a simpler model with fewer parameters but maintained predictive value. Such a mortality prediction model could facilitate future studies and performance monitoring.

## Methods

### Study design and setting

This study was a retrospective multicentre observational study including adult patients fulfilling the Sepsis-3 criteria admitted to one of four mixed surgical and medical ICUs between 2015 and 2018. This study did not include patients admitted to specialised ICUs (cardiothoracic or neurosurgical). The Strengthening the Reporting of Observational Studies in Epidemiology (STROBE) guidelines were followed.^16^

### Participants

Patients fulfilling the Sepsis-3 criteria with a total SOFA score of 2 or more on ICU admission were included. All admitted patients had an assumed baseline SOFA score of 0. To establish the presence of suspected infection, included admissions had to fulfil the following criteria: At least one blood culture collected within 24 h of ICU admission and antibiotics administrated within 24 h before to 72 h after blood culture collection. These criteria were in accordance with the Sepsis-3 guidelines.^4^ Patients directly transferred from another ICU, planned ICU admissions following elective surgery and patients with cardiac arrest within 6 h prior, to 1 h after ICU admission were excluded.

### Data sources

Data collection was performed by multiple trained data collectors using standardised and clearly defined guidelines. Data such as comorbidities, physiological parameters and laboratory findings were collected from electronic medical records. In addition, SOFA parameters, survival data, age and sex were collected from the Patient Administrative System for Intensive Care Units (PASIVA).

### Variables

The worst recorded value 6 h prior, to 1 h after ICU admission was documented regarding physiological and laboratory parameters. SOFA and SAPS-3 scores were calculated based on the worst recorded values 1 h prior, to 1 h after ICU admission. Immunosuppression was defined as immunosuppressive treatment such as chemotherapy or neutropaenia with absolute neutrophil count <1.5 × 10^9^/L before ICU admission. Corticosteroid treatment was only recorded for a treatment duration of more than 3 days before ICU admission. Septic shock was defined as vasopressor use and lactate >2 mmol/L at ICU admission. The patients were divided into two subgroups based on the outcome: Alive more than 30 days after ICU admission (survivors) and dead within 30 days from ICU admission (non-survivors). We used the Swedish 2016 SAPS-3 calibration, which estimates the 30-day mortality, to calculate the estimated mortality ratio (EMR).^17^ Therefore, we used 30-day mortality as the mortality measure when dividing the patients.

### Statistics

The two-sample Wilcoxon rank-sum test (Mann-Whitney U-test) was used to test for a difference in the continuous variable’s median. The two-proportions Z-test (Chi-squared test) was used to test for a difference in proportions. *p*-Values of less than 0.05 were considered significant. Backward stepwise multivariate logistic regression was performed with 30-day mortality as the outcome and the parameters with the highest significance from the univariate logistic regression as factors. This method begins with a complete model including several variables and then removes one variable after another to create a reduced model with the most significant independent variables. The area under the receiver operating characteristic (ROC) curve (AUC) was used to assess the predictive power of our models. Differences in AUC were tested with a method by DeLong et al.^18^

### Ethics

This study was approved by a Regional Ethical Review Board and conformed to the Declaration of Helsinki.

## Results

### Participants

A total of 7556 ICU admissions were identified, where 2223 admissions fulfilled the inclusion criteria, 222 admissions were excluded, and 17 patients were lost to follow-up. Therefore, the total number of patients included in the study was 1984.

### Descriptive statistics

The 30-day mortality was 25.3%. Survivors were younger than non-survivors (*p* < 0.001). Septic shock was more common in non-survivors than survivors (*p* < 0.001). Survivors were likelier to have a higher body mass index (BMI) than non-survivors (*p* = 0.028). No difference between the sexes regarding 30-day mortality could be found. The median SAPS-3 score at ICU admission was 63 for survivors and 74 for non-survivors. The median SOFA score at ICU admission was seven for survivors and nine for non-survivors. See Table 1.

**Table 1.** Characteristics of the studied patient population.

Parameter number	All 1984 (100%)	Alive >30 days	Deceased ⩽30 days	*p*- Value*	Missing (%)
		1482 (74.7%)	502 (25.3%)		
General characteristics					
Age, years	69 (59–76)	68 (57–75)	72 (64–78)	<0.001	0
Male sex, %	58.1	57.9	58.6	0.83	0
BMI, kg/m^2^	26.6 (23.2–30.9)	26.8 (23.4–31.2)	26.0 (22.8–30.2)	0.028	21
Septic shock, %	39.5	36.5	48.2	<0.001	0
Morbidity measures					
SAPS-3 score at admission	65 (57–75)	63 (55–71)	74 (65–82)	<0.001	0
EMRSAPS-3 Swe, %	28	24	47	<0.001	0
EMRSAPS-3, %	46	42	64	<0.001	0
SOFA score at admission	7 (5–10)	7 (5–9)	9 (6–12)	<0.001	1
Comorbidities					
Immunosuppression, %	12.2	10.5	17.1	<0.001	0
Corticosteroids, %	14.3	13.0	18.3	0.0038	0
Diabetes Mellitus, %	24.2	24.2	24.3	1.00	0
Cardiovascular disease, %	46.0	43.9	52.2	0.0013	0
Ischaemic heart disease, %	19.0	19.0	19.1	0.99	0
Peripheral vascular disease, %	6.9	5.7	10.2	0.001	0
Atrial fibrillation, %	18.2	17.4	20.5	0.14	0
Heart failure, %	15.3	13.6	20.1	<0.001	0
Stroke/TIA, %	12.3	11.4	14.9	0.045	0
Asthma/COPD, %	19.1	20.5	14.9	0.0075	0
Liver disease, %	5.8	5.1	7.8	0.037	0
Renal disease, %	11.3	10.7	13.3	0.17	0
Malignancy, %	13.7	12.6	16.9	0.016	0
Physiological parameters					
Heart rate, bpm	115 (96–130)	114 (97–130)	116 (95–134)	0.32	0.6
Systolic blood pressure, mmHg	100 (79–124)	100 (80–126)	92 (75–120)	<0.001	1.7
Respiratory rate, /min	30 (24–38)	30 (23–37)	31 (24–40)	<0.001	3.9
GCS, mean (±1 SD)	12.3 (±3.57)	12.4 (±3.49)	11.8 (±3.78)	<0.001	3.7
SpO2, %	89 (80–95)	90 (80–95)	88 (80–93)	0.029	0.7
Body temperature, °C	37.5 (36.3–38.5)	37.6 (36.5–38.6)	37.0 (36.0–38.3)	<0.001	1.6
Vasopressor support, %	37.6	25.3	44.2	<0.001	0
Biochemistry					
CRP, mg/L	105 (33–224)	93 (29–212)	136 (52–265)	<0.001	7.1
WBC, 10^9^/L	13.8 (8.7–19.9)	13.8 (8.8–19.9	13.9 (8.5–20.0)	0.91	0.5
Platelet count, 10^9^/L	208 (140–292)	213 (145–296)	197 (115–284)	<0.001	6
Lactate, mmol/L	2.7 (1.4–5.1)	2.6 (1.4–4.8)	3.3 (1.7 –5.8)	<0.001	3.6
APTT	30 (27–36)	30 (26–35)	31 (28–41)	0.0021	61
Creatinine, µmol/L	116 (76–207)	110 (74–188)	148 (84–252)	<0.001	3.2
Bilirubin, µmol/L	11 (7–20)	11 (7–19)	13 (8–26)	<0.001	6.5
pH	7.3 (7.2–7.4)	7.3 (7.2–7.4)	7.3 (7.2–7.4)	0.015	53

BMI: body mass index; SAPS: Simplified Acute Physiology Score; EMR: estimated mortality rate; SOFA: Sequential Organ Failure Assessment; TIA: transient ischaemic attack; COPD: chronic obstructive pulmonary disease; GCS: Glasgow Coma Scale; SpO_2_: peripheral oxygen saturation; CRP: C-reactive protein; WBC: white blood cell count; APTT: activated partial thromboplastin time; pH: hydrogen ion concentration.

Values presented as medians and interquartile range or percentages, with the exception of GCS, which is presented as means and standard deviations (SD).

* Calculated using the two-samples Wilcoxon rank-sum test (Mann-Whitney U-test) and the two-proportions Z-test (Chi-squared test) as appropriate.

Our analyses found almost all documented comorbidities to be more frequent in non-survivors than survivors, except for asthma or chronic obstructive pulmonary disease (COPD), which was more common among survivors than non-survivors. Only 14.9% of patients in the non-survivor group had a diagnosis of asthma or COPD, compared to 20.5% of patients in the survivor group (*p* = 0.0075). The prevalence of documented comorbidities did not differ between survivors and non-survivors regarding diabetes mellitus (type I and II), ischaemic heart disease, atrial fibrillation and renal disease. See Table 1.

Non-survivors had lower systolic blood pressure, higher respiratory rate, lower Glasgow Coma Scale (GCS) score, lower peripheral oxygen saturation (SpO_2_) and lower body temperature than survivors. A higher percentage of non-survivors than survivors needed vasopressor support (44.2% and 25.3%, respectively). There was no difference in heart rate between the two groups (*p* = 0.32). Non-survivors had higher C-reactive protein (CRP) levels, lower platelet counts, higher lactate levels, higher creatinine levels and higher bilirubin levels. No difference was observed between the two groups regarding white blood cell count (WBC). See Table 1.

### Multivariate logistic regression

**Figure 1.**
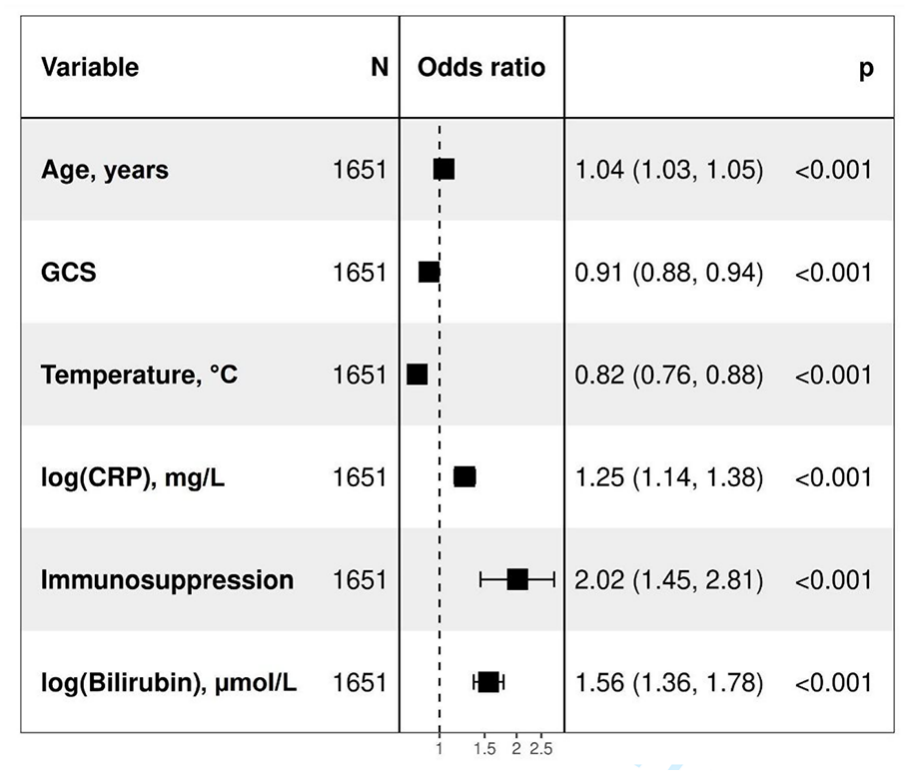
Multivariate logistic regression of a simple six-parameter model consisting of age, immunosuppression, Glasgow Coma Scale, body temperature, C-reactive protein and bilirubin in predicting 30-day mortality in sepsis patients admitted to the ICU. ICU: intensive care unit; GCS: Glasgow Coma Scale; CRP: C-reactive protein.

Out of the 36 parameters in Table 1, 24 were significant in univariate logistic regression analyses (see Supplemental Material). Subsequently, these 24 were used in a backward stepwise multivariate logistic regression analysis. Several simpler models were created using backward stepwise multivariate logistic regression, each including four to six variables, and the simple model with the highest AUC was chosen. A six-parameter model was found with odds ratios (OR) of more than one for age, immunosuppression, CRP and bilirubin and OR of less than one for GCS score and body temperature. See Figure 1.

**Figure 2.**
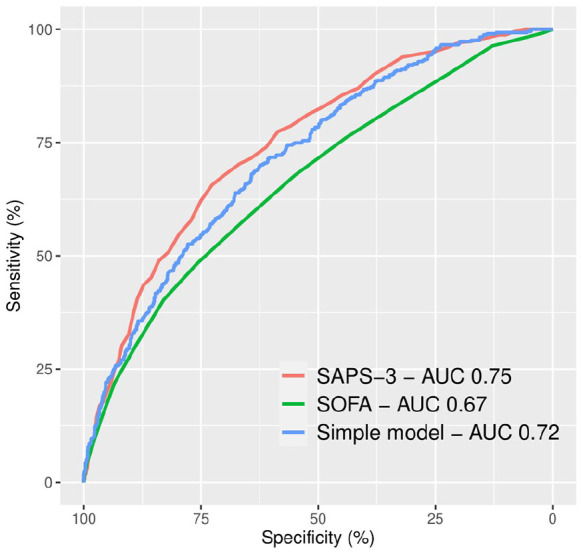
ROC curves and AUC for SAPS-3, SOFA score, and a simple six-parameter model consisting of age, immunosuppression, Glasgow Coma Scale, body temperature, C-reactive protein and bilirubin in predicting 30-day mortality in sepsis patients admitted to the ICU. ROC: receiver operating characteristic; AUC: area under curve; SAPS: Simplified Acute Physiology Score; SOFA: Sequential Organ Failure Assessment; ICU: intensive care unit.

SAPS-3, on ICU admission, had an AUC of 0.75 (95% CI 0.72–0.77). Our six-parameter model had an AUC of 0.72 (95% CI 0.69–0.75) with no difference in predictive strength compared to SAPS-3 (*p* = 0.071). SOFA score upon ICU admission had an AUC of 0.67 (95% CI 0.64–0.70). Both SAPS-3 and our model had a better predictive value than the SOFA score (*p* < 0.001 and *p* = 0.0019, respectively). See Figure 2.

Internal validation of the six-parameter model was undertaken through five-fold cross-validation with 50 repeats, which yielded an AUC of 0.71 (95% CI 0.66–0.76).

## Discussion

SAPS-3 is a well-known and widely used model in ICUs worldwide. SAPS-3 is well-studied in general ICU populations and has relatively high sensitivity and specificity, with several studies showing an AUC of 0.80–0.85.^8–11^ This is further supported by a recent study in a large general ICU population, which showed an AUC of 0.85 for SAPS-3.^19^ However, SAPS-3 is not explicitly designed for sepsis patients, and the discriminative power of SAPS-3 was notably lower in our analyses in a sepsis population, with an AUC of 0.75. Thus, our findings suggest that the performance of SAPS-3 in a sepsis population is lower than in a general ICU population, which is important since sepsis is common in the ICU.^1,2^ SAPS-3 also requires extensive clinical information, which limits its use. Another potential mortality prediction model for sepsis patients in the ICU is the SOFA score. The SOFA score has previously been shown to predict hospital mortality in ICU patients adequately.^20^ Like our model, it also consists of six parameters and is thus simpler than SAPS-3. Both the SOFA score and our model include the GCS score and bilirubin levels. However, the SOFA score did not perform well in this study compared to our model and SAPS-3. The SOFA score is not mainly intended for mortality prediction but rather to estimate and monitor the degree of organ dysfunction. One could argue that the SOFA score complements mortality prediction tools rather than replaces them.^15^

We found a simple six-parameter model that was as good as SAPS-3 in the Sepsis-3 population in our large cohort. This model suggests a higher risk of death within 30 days of ICU admission with advanced age, immunosuppression, low GCS score, low body temperature, increased CRP level and increased bilirubin level. It combines measures of systemic inflammation (CRP), immune dysregulation (normo- or hypothermia during sepsis) and organ dysfunction (GCS score and bilirubin). Finding age and immunosuppression to be important prognostic factors was no surprise. This model is more straightforward and takes less time to document than SAPS-3, which contains 20 parameters. The parameters needed to predict 30-day mortality with this model are usually recorded at admission and daily on patients with sepsis in the ICU. Only immunosuppression is needed from the medical history. It includes only two laboratory values and does not require blood gas analysis.

Additionally, fewer parameters also minimise the risk of error. Since sepsis accounts for around a third of all ICU admissions, a simple predictive model for mortality adapted for patients with sepsis is relevant. Such a model would benefit low- and middle-income countries (LMIC) ICUs. Common challenges for ICU prognostic models in LMICs are a lack of laboratory testing resources and a lack of staff training and availability, which results in missing or inaccurate parameter values.^21^ A model containing only six parameters would facilitate quick and correct documentation. Both CRP and bilirubin can be analysed relatively rapidly and cheaply. Age, immunosuppression, GCS score and body temperature can all be obtained bedside quickly.

The association between low or normal body temperature and increased mortality in sepsis is reflected in SAPS-3 and supported by several studies showing that hypo- and normothermia in patients with sepsis requiring intensive care increase mortality.^22–24^ As with SAPS-3 and SOFA, our model also includes bilirubin. Previous studies have found that elevated bilirubin levels at ICU admission are an early independent predictor of mortality in patients with sepsis.^25,26^ Interestingly, our analyses suggest that CRP and lactate have important prognostic values in sepsis. Both CRP and lactate are variables that are routinely analysed but not included in any standard mortality prediction model in the ICU. We found elevated CRP levels to be prognostic and thus included it in our simplified model. In a separate publication, our research group previously explored the finding that CRP is a strong independent prognostic marker for 30-day mortality.^27^ Elevated lactate levels were strongly correlated with 30-day mortality in this study’s univariate logistic regression results, and similar results can be seen in previous studies.^28,29^ Though, lactate levels did not add enough independent predictive power to be included in our simple model. Lactate levels have been reported to be a good predictor of mortality in sepsis and have good discriminative power, with an AUC of 0.66.^30^ Although lactate was not included in the simple prognostic model of this study, further research on the predictive value of lactate levels is of interest.

In contrast, our analyses showed no significant difference in WBC between survivors and non-survivors. WBC is included in SAPS-3, and although it is clinically used to follow the course of an infection, the prognostic value of WBC in sepsis is poorly studied. One prior study has indicated that WBC is not of predictive value in sepsis patients.^31^ Likewise, while tachycardia is included in SAPS-3, heart rate was not shown to correlate with 30-day mortality in our analyses. Although the correlation between heart rate and mortality in sepsis is poorly studied, some studies have suggested that tachycardia and relative tachycardia (the ratio between heart rate and body temperature) are associated with increased mortality in sepsis.^32,33^

Since we did not have access to data on baseline SOFA scores and the Sepsis-3 criteria include an acute SOFA score increase of two points or more, we assumed all admitted patients to have a baseline SOFA score of 0. This means that patients with chronic organ failure and a baseline SOFA score of 1 or more may have been falsely categorised as having sepsis due to the study design. The relatively high median SOFA score in our results implies that most patients would likely have had an acute SOFA score increase of at least two points anyway.

Our results noted a higher prevalence of asthma/COPD in survivors than non-survivors. Exacerbations of COPD may be clinically challenging to distinguish from sepsis. Furthermore, COPD patients also tend to obtain antibiotic treatment more liberally than others, and antibiotic treatment 24 h prior, to 72 h after ICU admission was one of the inclusion criteria. Thus, it is possible that some patients with COPD were falsely included as septic patients in this study. Since COPD patients admitted to the ICU because of respiratory failure have relatively low mortality, this may explain an overrepresentation of asthma/COPD in survivors.^34^ Another minor limitation in this study was the lack of documentation of diabetes type. According to earlier publications, patients with type 2 diabetes have increased mortality in sepsis.^35,36^ Our study showed no such increase, perhaps because the two types of diabetes were not distinguished in the documentation process.

This study was conducted at multiple centres and included a relatively large patient sample, strengthening our results. Furthermore, the two databases used for data collection were not explicitly designed for this study but had multiple purposes, making them less susceptible to confirmation bias. Several data collectors documented data using one standardised and clearly defined guideline, which reduced the risk of systematic errors. However, it is worth noting that the AUC of our simple six-parameter model, like the AUC for SAPS-3 in this study, indicates only a moderately performing model. Our model needs external validation in other Sepsis-3 cohorts in the ICU before its utility can be confirmed.

Furthermore, this six-parameter model can only be used on a group level, not to estimate individual patients’ mortality risk.

Further research on the calibration of this model is needed to make the mortality prediction of individual patients possible, which is necessary to make this model an equal alternative to SAPS-3. Simple and accurate risk-adjustment models are important for easy but accurate quality control of sepsis care and the design of simple yet robust studies on sepsis in intensive care. Our suggested six-parameter model is undoubtedly a good candidate for such a model.

## Conclusion

Although sepsis is very common in the ICU, SAPS-3 has a lower prognostic value in ICU admissions fulfilling the Sepsis-3 criteria than in the general ICU population. SOFA score upon ICU admission has poorer performance than SAPS-3 but was not designed with mortality prediction in mind. A simpler six-parameter method using age, immunosuppression, GCS score, body temperature, CRP and bilirubin on admission predicts 30-day mortality upon ICU admission for sepsis patients just as well as SAPS-3. These findings could allow the design of studies with access to less data without compromising quality and facilitate performance monitoring.

## Supplemental Material

sj-docx-1-inc-10.1177_17511437221149572 – Supplemental material for A simple mortality prediction model for sepsis patients in intensive care Click here for additional data file. Supplemental material, sj-docx-1-inc-10.1177_17511437221149572 for A simple mortality prediction model for sepsis patients in intensive care by Hazem Koozi, Adina Lidestam, Maria Lengquist, Patrik Johnsson and Attila Frigyesi in Journal of the Intensive Care Society

## References

[1] Lengquist M Lundberg OHM Spångfors M , et al. Sepsis is underreported in Swedish intensive care units: a retrospective observational multicentre study. Acta Anaesthesiol Scand 2020; 64: 1167–1176. 32463121 10.1111/aas.13647

[2] Vincent JL Lefrant JY Kotfis K , et al. Comparison of European ICU patients in 2012 (ICON) versus 2002 (SOAP). Intensive Care Med 2018; 44: 337–344. 29450593 10.1007/s00134-017-5043-2 PMC5861160

[3] Rudd KE Johnson SC Agesa KM , et al. Global, regional, and national sepsis incidence and mortality, 1990-2017: analysis for the Global Burden of Disease Study. Lancet 2020; 395: 200–211. 31954465 10.1016/S0140-6736(19)32989-7 PMC6970225

[4] Singer M Deutschman CS Seymour CW , et al. The third international consensus definitions for sepsis and septic shock (Sepsis-3). JAMA 2016; 315: 801–810. 26903338 10.1001/jama.2016.0287 PMC4968574

[5] Vincent JL Moreno R. Clinical review: scoring systems in the critically ill. Crit Care 2010; 14: 207. 20392287 10.1186/cc8204 PMC2887099

[6] Metnitz PG Moreno RP Almeida E , et al. SAPS 3–from evaluation of the patient to evaluation of the intensive care unit. Part 1: Objectives, methods and cohort description. Intensive Care Med 2005; 31: 1336–1344. 16132893 10.1007/s00134-005-2762-6 PMC1315314

[7] Falcão ALE Barros AGA Bezerra AAM , et al. The prognostic accuracy evaluation of SAPS 3, SOFA and APACHE II scores for mortality prediction in the surgical ICU: an external validation study and decision-making analysis. Ann Intensive Care 2019; 9: 18. 30701392 10.1186/s13613-019-0488-9 PMC6353976

[8] Keegan MT Gajic O Afessa B. Comparison of APACHE III, APACHE IV, SAPS 3, and MPM0III and influence of resuscitation status on model performance. Chest 2012; 142: 851–858. 22499827 10.1378/chest.11-2164 PMC3465106

[9] Ledoux D Canivet JL Preiser JC , et al. SAPS 3 admission score: an external validation in a general intensive care population. Intensive Care Med 2008; 34: 1873–1877. 18592214 10.1007/s00134-008-1187-4

[10] Ma QB Fu YW Feng L , et al. Performance of simplified acute physiology score 3 in predicting hospital mortality in Emergency Intensive Care Unit. Chin Med J 2017; 130: 1544–1551. 28639569 10.4103/0366-6999.208250 PMC5494917

[11] Metnitz B Schaden E Moreno R , et al. Austrian validation and customization of the SAPS 3 admission score. Intensive Care Med 2009; 35: 616–622. 18846365 10.1007/s00134-008-1286-2

[12] Mbongo CL Monedero P Guillen-Grima F , et al. Performance of SAPS3, compared with APACHE II and SOFA, to predict hospital mortality in a general ICU in Southern Europe. Eur J Anaesthesiol 2009; 26: 940–945. 19606046 10.1097/EJA.0b013e32832edadf

[13] Basile-Filho A Lago AF Menegueti MG , et al. The use of SAPS 3, SOFA, and Glasgow coma scale to predict mortality in patients with subarachnoid hemorrhage: a retrospective cohort study. Medicine 2018; 97: e12769. 10.1097/MD.0000000000012769 PMC6203557 30313090

[14] Raith EP Udy AA Bailey M , et al. Prognostic accuracy of the SOFA Score, SIRS criteria, and qSOFA score for In-hospital mortality among adults with suspected infection admitted to the Intensive Care Unit. JAMA 2017; 317: 290–300. 28114553 10.1001/jama.2016.20328

[15] Vincent JL Moreno R Takala J , et al. The SOFA (Sepsis-related organ failure assessment) score to describe organ dysfunction/failure. On behalf of the Working Group on Sepsis-Related Problems of the European Society of Intensive Care Medicine. Intensive Care Med 1996; 22: 707–710. 8844239 10.1007/BF01709751

[16] Cuschieri S. The STROBE guidelines. Saudi J Anaesth 2019; 13: S31–S34. 30930717 10.4103/sja.SJA_543_18 PMC6398292

[17] Nordlund P Walther S. Riskjusteringsmodeller inom svensk intensivvård, https://www.icuregswe.org/globalassets/publikationer/fokusrapporter/fokusrapport_riskjustering_i_sir.pdf (2019, accessed 10 October 2019).

[18] DeLong ER DeLong DM Clarke-Pearson DL. Comparing the areas under two or more correlated receiver operating characteristic curves: a nonparametric approach. Biometrics 1988; 44: 837–845. 3203132

[19] Holmgren G Andersson P Jakobsson A , et al. Artificial neural networks improve and simplify intensive care mortality prognostication: a national cohort study of 217,289 first-time intensive care unit admissions. J Intensive Care 2019; 7: 44. 31428430 10.1186/s40560-019-0393-1 PMC6697927

[20] Ferreira FL Bota DP Bross A , et al. Serial evaluation of the SOFA score to predict outcome in critically ill patients. JAMA 2001; 286: 1754–1758. 11594901 10.1001/jama.286.14.1754

[21] Haniffa R Isaam I De Silva AP , et al. Performance of critical care prognostic scoring systems in low and middle-income countries: a systematic review. Crit Care 2018; 22: 18. 29373996 10.1186/s13054-017-1930-8 PMC5787236

[22] Kushimoto S Gando S Saitoh D , et al. The impact of body temperature abnormalities on the disease severity and outcome in patients with severe sepsis: an analysis from a multicenter, prospective survey of severe sepsis. Crit Care 2013; 17: R271. 24220071 10.1186/cc13106 PMC4057086

[23] Lee B Inui D Suh G , et al. Association of body temperature and antipyretic treatments with mortality of critically ill patients with and without sepsis: multi-centered prospective observational study. Crit Care 2012; 16: R33. 22373120 10.1186/cc11211 PMC3396278

[24] Young PJ Saxena M Beasley R , et al. Early peak temperature and mortality in critically ill patients with or without infection. Intensive Care Med 2012; 38: 437–444. 10.1007/s00134-012-2478-3 22290072

[25] Zhai R Sheu CC Su L , et al. Serum bilirubin levels on ICU admission are associated with ARDS development and mortality in sepsis. Thorax 2009; 64: 784–790. 19482841 10.1136/thx.2009.113464 PMC2735615

[26] Patel JJ Taneja A Niccum D , et al. The association of serum bilirubin levels on the outcomes of severe sepsis. J Intensive Care Med 2015; 30: 23–29. 23753252 10.1177/0885066613488739

[27] Koozi H Lengquist M Frigyesi A. C-reactive protein as a prognostic factor in intensive care admissions for sepsis: a Swedish multicenter study. J Crit Care 2020; 56: 73–79. 31855709 10.1016/j.jcrc.2019.12.009

[28] Villar J Short JH Lighthall G. Lactate predicts both short- and long-term mortality in patients with and without sepsis. Infect Dis 2019; 12: 1178633719862776. 10.1177/1178633719862776 PMC6686323 31431799

[29] Thomas-Rueddel DO Poidinger B Weiss M , et al. Hyperlactatemia is an independent predictor of mortality and denotes distinct subtypes of severe sepsis and septic shock. J Crit Care 2015; 30: 439.e1–439.e6. 10.1016/j.jcrc.2014.10.027 25466313

[30] Liu Z Meng Z Li Y , et al. Prognostic accuracy of the serum lactate level, the SOFA score and the qSOFA score for mortality among adults with sepsis. Scand J Trauma Resusc Emerg Med 2019; 27: 51. 31039813 10.1186/s13049-019-0609-3 PMC6492372

[31] Suberviola B Castellanos-Ortega A González-Castro A , et al. [Prognostic value of procalcitonin, C-reactive protein and leukocytes in septic shock]. Med Intensiva 2012; 36: 177–184. 22055776 10.1016/j.medin.2011.09.008

[32] Parker MM Shelhamer JH Natanson C , et al. Serial cardiovascular variables in survivors and nonsurvivors of human septic shock: heart rate as an early predictor of prognosis. Crit Care Med 1987; 15: 923–929. 3652707 10.1097/00003246-198710000-00006

[33] Leibovici L Gafter-Gvili A Paul M , et al. Relative tachycardia in patients with sepsis: an independent risk factor for mortality. QJM 2007; 100: 629–634. 17846061 10.1093/qjmed/hcm074

[34] Gadre SK Duggal A Mireles-Cabodevila E , et al. Acute respiratory failure requiring mechanical ventilation in severe chronic obstructive pulmonary disease (COPD). Medicine 2018; 97: e0487. 10.1097/MD.0000000000010487 PMC5944543 29703009

[35] Frydrych LM Bian G O’Lone DE , et al. Obesity and type 2 diabetes mellitus drive immune dysfunction, infection development, and sepsis mortality. J Leukoc Biol 2018; 104: 525–534. 30066958 10.1002/JLB.5VMR0118-021RR

[36] Tiwari S Pratyush DD Gahlot A , et al. Sepsis in diabetes: a bad duo. Diabetes Metab Syndr 2011; 5: 222–227. 25572769 10.1016/j.dsx.2012.02.026

