# Supplementary material for: A simple mortality prediction model for sepsis patients in intensive care
Source: J Intensive Care Soc. 2023 Feb 1;24(4):372–8. doi: 10.1177/17511437221149572 (PMC10572475; doi:10.1177/17511437221149572)
Supplement: sj-docx-1-inc-10.1177_17511437221149572 – Supplemental material for A simple mortality prediction model for sepsis patients in intensive care [file sj-docx-1-inc-10.1177_17511437221149572.docx]

**Supplementary table** **- Univariate logistic regression analysis of several parameters in predicting 30-day mortality in sepsis patients admitted to the ICU.**

| Parameter | OR (95% CI) | *p* level | Missing (%) |
| --- | --- | --- | --- |
| Age, years | 1.03 (1.02–1.04) | **** | 0 |
| BMI, kg/m^2^ | 0.98 (0.96–1.00) | * | 21 |
| Immunosuppression, yes/no | 1.76 (1.32–2.34) | *** | 0 |
| Corticosteroids, yes/no | 1.51 (1.14–1.97) | ** | 0 |
| Cardiovascular disease, yes/no | 1.40 (1.14–1.72) | ** | 0 |
| Peripheral vascular disease, yes/no | 1.86 (1.29–2.66) | *** | 0 |
| Heart failure, yes/no | 1.60 (1.22–2.07) | *** | 0 |
| Stroke/TIA, yes/no | 1.36 (1.01–1.82) | * | 0 |
| Asthma/COPD, yes/no | 0.68 (0.51–0.89) | ** | 0 |
| Liver disease, yes/no | 1.56 (1.04–2.31) | * | 0 |
| Malignancy, yes/no | 1.42 (1.07–1.88) | * | 0 |
| Systolic blood pressure, mmHg | 0.99 (0.99–1.00) | *** | 1.7 |
| Respiratory rate, /min | 1.02 (1.01–1.03) | ** | 3.9 |
| GCS | 0.95 (0.93–0.98) | *** | 3.7 |
| SpO_2_ , %  Body temperature, °C | 1.00 (0.99–1.01)  0.85 (0.80–0.90) | *ns* **** | 0,7  1.6 |
| Vasopressor support, yes/no | 1.48 (1.20–1.83) | *** | 0 |
| CRP, mg/L | 1.00 (1.00–1.00) | **** | 7.1 |
| Platelet count, 10^9^/L | 1.00 (1.00–1.00) | ** | 6.0 |
| Lactate, mmol/L | 1.08 (1.05–1.11) | **** | 3.6 |
| APTT | 1.02 (1.01–1.04) | *** | 61 |
| Creatinine, *µ*mol/L | 1.00 (1.00–1.00) | * | 3.2 |
| Bilirubin, *µ*mol/L | 1.01 (1.01–1.01) | **** | 6.5 |
| pH | 0.24 (0.09–0.66) | ** | 53 |

* *<*0.05, ** *<*0.01, *** *<*0.001, **** *<*0.0001, ns (non-significant) *≥*0.05. ICU, intensive care unit. OR, odds ratio. CI, confidence interval. BMI, body mass index. TIA, transient ischemic attack. COPD, chronic obstructive pulmonary disease. GCS, Glasgow Coma Scale. SpO_2_, peripheral oxygen saturation. CRP, C-reactive protein. APTT, activated partial thromboplastin time. pH, hydrogen ion concentration.
